# Assessment of potential habitat suitability for kiwifruit (*Actinidia* spp) in Shaanxi Province under climate change scenarios

**DOI:** 10.3389/fpls.2025.1617802

**Published:** 2025-09-01

**Authors:** Zhen Niu, Dong Han, Zijie Niu, Petros A. Roussos, Tao Cheng, Jiyuan Xie, Geni Cao, Dandan Liu, Ning Jin, Dongyan Zhang

**Affiliations:** ^1^ College of Mechanical and Electronic Engineering, Northwest A&F University, Yangling, China; ^2^ Shaanxi Key Laboratory of Agricultural Information Perception and Intelligent Service, Northwest A&F University, Yangling, China; ^3^ Laboratory of Pomology, Department of Crop Science, Agricultural University of Athens, Athens, Greece; ^4^ Shaanxi Fruit Tree Science Research Institute, Shaanxi Fruit Industry group Company Limited, Yangling, China; ^5^ Department of Resources and Environmental Engineering, Shanxi Institute of Energy, Jinzhong, China

**Keywords:** kiwifruit, climate change, habitat suitability, SSP, MaxEnt

## Abstract

**Introduction:**

Climate change and anthropogenic activities have significantly altered the distribution patterns of kiwifruit resources in Shaanxi Province. Clarifying the characteristics of future climate suitability zones can provide scientific support for optimizing industrial planning and mitigating meteorological disaster risks.

**Methods:**

This study applied the MaxEnt model with ENMeval package parameter optimization. Environmental factor contributions were quantified via jackknife tests, and spatial consistency between major producing counties and ecological suitability areas was analyzed using ArcGIS. The dynamic shifts and centroid migration patterns of kiwifruit habitats under four Shared Socioeconomic Pathways (SSP) climate scenarios (SSP126, SSP245, SSP370, SSP585) during the 2050–2070 were simulated.

**Results and discussion:**

Currently, high-suitability zones (13,100 km², 6.38% of the province) are concentrated in the northern Qinling Mountains, Weihe River terrace irrigation areas, and the Danjiang/Hanjiang River basins of southern Shaanxi, while moderate-suitability zones cover 32,600 km² (15.86%). Critical environmental drivers include the mean temperature of the coldest quarter, elevation, annual temperature variation range, and soil moisture content. Future projections reveal substantial reductions in high-suitability habitats (SSP126: 79.12% decrease to 2,700 km² by 2080; SSP245/370/585 reductions of 58.01%/68.33%/61.70%), intensified habitat fragmentation, and slight northwestward centroid shift. This study systematically evaluates climate change impacts on kiwifruit suitability zones, offering a theoretical basis for intensive adjustments in Shaanxi’s cultivation zones, agricultural policy formulation, and biodiversity conservation strategies.

## 1 Introduction

Kiwifruit (*Actinidia* spp) is a perennial deciduous vine known for its sweet and tangy flavor. It is rich in vitamin C, dietary fiber, and essential minerals, earning it the title of the “king of fruits” (Han et al., 2023). Moreover, it is notable for its medicinal properties, including its ability to clear heat and water, act as an antioxidant, and regulate blood sugar levels (Kaur et al., 2022). Shaanxi Province is a major global kiwifruit producer, with an output of 1.463 million tonnes in 2023. However, climate change and human activities have disrupted kiwifruit’s natural habitat. As its climatic niche becomes less suitable, kiwifruit cultivation is shifting to more favorable regions (Li et al., 2015). The response of vegetation to rising temperatures is not only related to carbon cycle processes, but also to a wide range of areas, including agricultural productivity, food security and public health (Wang et al., 2021; Wang and Wang, 2023).

Global temperatures have risen significantly in recent decades, with climate change progressing faster than previously predicted (Lynn and Peeva, 2021). In China, temperature trends closely mirror global patterns, affecting agriculture and crop distributions (Chen and Gong, 2021). Climate change alters species distributions, sometimes leading to irreversible ecosystem shifts (Priya et al., 2023). Since agricultural productivity depends on climatic conditions, understanding how climate affects kiwifruit habitat is crucial (Smith et al., 2017). In the context of a changing climate, the suitable growing areas for plants will also change, making it particularly important to predict the impact of climate change on plant distribution patterns. Research on kiwifruit species is now focused on molecular biology, horticulture, plant protection, harvesting and post-harvest handling, food science and technology, and pharmacology and phytochemistry. Nevertheless, relatively few studies have been conducted on the ecological conservation predictions. In the context of rising global average temperatures, kiwifruit premature senescence syndrome, marked by the sudden collapse and death of affected plants, is becoming more prevalent and pervasive. This phenomenon can be considered a consequence of climate change, as highlighted in research conducted by Rajan. (Rajan et al., 2024) High temperature stress exerts a direct effect on the growth, physiology and partitioning of photosynthetic products in kiwifruit plants. In addition, it has been demonstrated that high temperature stress can lead to soil hypoxia, albeit in an indirect manner. At critical stages of kiwifruit development, elevated temperatures result in the redistribution of resources towards nutrient growth, leading to a significant reduction in carbohydrate and vitamin C levels (Richardson et al., 2004). Furthermore, excessive flooding hinders the exchange of air between the soil and the atmosphere, resulting in limited oxygen utilization by the plant root system, which in turn inhibits root respiration and reduces root activity. Furthermore, during periods of flooding, plant leaf stomata typically remain closed, resulting in chlorophyll degradation, which in turn affects gas exchange and photosynthetic rates, consequently leading to a decline in crop quantity and quality (Huo et al., 2022). The growth cycle of crops, yield formation and their suitable growing areas will be significantly altered by increasing temperatures and changes in precipitation patterns (Bokor et al., 2021). The growth of kiwifruit is strongly influenced by climate, soil and topographic conditions, and kiwifruit quality and yield can be optimized when suitable ecological conditions are present in the growing area (Gao et al., 2022). Elucidation of the distribution pattern of kiwifruit and its response to climate change can facilitate kiwifruit industry planning and reduce meteorological disaster winds.

Species Distribution Models (SDMs) are mathematical or statistical models that analyze the relationship between species and environmental factors. They are constructed based on specific algorithms that integrate field observations of species (species occurrence records or distribution data) and environmental factors (climate, topography, soil, vegetation cover, etc.). The fundamental principle of species distribution modelling is predicated on the theory of ecological niches, which posits that each species possesses its own unique ecological requirements and range of adaptation to the environment. Consequently, by analyzing the environmental characteristics of a species’ location, the model can identify significant effects of environmental factors on the species’ distribution and predict its potential distribution in other areas. The model outputs can be interpreted as the probability of existence of a species, the abundance of a species population, or the habitat suitability of a species, etc (Elith and Leathwick, 2009; Yao et al., 2025). In recent years, with the increasing application of big data such as global climate interpolated data, terrestrial remote sensing data and soil attribute data, it provides strong scientific support for the improvement of species distribution models. Species Distribution Models (SDMs) have undergone continuous methodological innovations. The Nested Species Distribution Model (N-SDM) has enhanced predictive robustness by mitigating niche truncation issues through multi-scale environmental variables (Guisan et al., 2025). Concurrent research emphasizes the necessity of optimizing model selection based on species-specific characteristics and ensuring predictive reliability through reproducible code (Valavi et al., 2022). Microclimate-scale models reveal that traditional macroclimate models may overestimate the threat of climate change to amphibian species (Stickley and Fraterrigo, 2023) while regional-scale applications, such as those in mangrove ecosystems, have validated the value of SDMs in conservation planning within dynamic environments (Samal et al., 2023). Current trends focus on the integration of multi-scale data and the improvement of model adaptability, yet the prediction accuracy for species with non-equilibrium distributions remains an area requiring significant advancement. The MaxEnt model has become one of the most widely used and most accurate models for species distribution modelling by virtue of the characteristics of low data requirement, high stability, strong predictive ability and easy interpretability (Zhang et al., 2024). Previous studies have shown the MaxEnt model demonstrated that suitable plant habitats for Chinese wind chamomile exhibit a migration in response to climate change, with future warming anticipated to result in a migration of vegetation to circumpolar latitudes and higher elevations (Zhao et al., 2024). In recent years, research teams have proposed agronomic management strategies for environmentally-matched ginseng species to promote carbon reduction and optimize productivity. These strategies were formulated by combining meta-analysis and MaxEnt models, with the aim of facilitating migration to higher latitudes (Yan et al., 2024). In addition, it has been demonstrated that the optimization of MaxEnt model parameters can significantly enhance its prediction accuracy and reliability (Phillips et al., 2006). At present, there have been many studies on the distribution of kiwifruit, including those at the national level (Wang et al., 2024), provincial level such as Sichuan, Guizhou, Liaoning (He et al., 2018), and county-level such as Meixian, Zhouzhi, etc. However, there is a lack of systematic research specifically at the provincial level in Shaanxi Province.

The SSP scenarios provide constraints from the dimension of socio-economic drivers for species distribution prediction by integrating variables such as population growth, technological innovation, and climate policies. In this study, the optimized MaxEnt model and ArcGIS software were utilized to simulate the potential distribution of kiwifruit species under different SSP scenarios (SSP126, SSP245, SSP370, SSP585) for the period 2040-2080, and to synthesize the contribution of the environmental factor factors and the significant value replacement. Knife cut tests were used to determine suitable intervals for the environmental factor factors and to quantify the potential geographic distribution and area of kiwifruit. The objectives were threefold: firstly, to classify the suitability of kiwifruit under current climatic conditions, and analyze the relationship between distribution areas and major environmental factors; secondly, to predict the distribution of kiwifruit habitats under future meteorological scenarios, and trends in area and center of mass; and thirdly, to verify the consistency between kiwifruit climatic suitability zones and yield, and analyze the relationship between yield and environmental factors in a multifactorial manner to predict future yield trends. In light of the findings of the climate suitability study, a large-scale and specialized planting pattern is to be formulated in order to promote the intensive development of the kiwifruit industry and enhance its overall efficiency.

## 2 Materials and methods

### 2.1 Research framework

**Figure 1:**
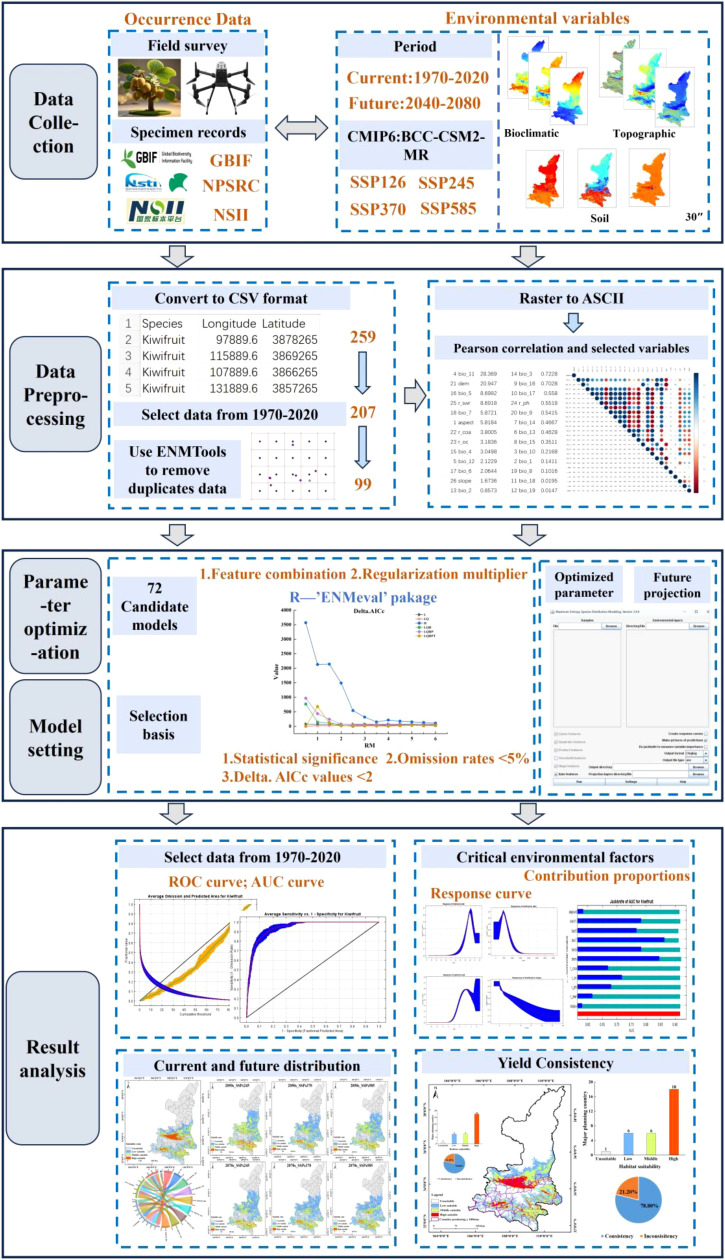
Technology road map.

Based on the research objectives and fundamentals of the optimized MaxEnt model, a framework was constructed to simulate the current and future suitable distributions of kiwifruit species (**Figure 1**). The framework is divided into four sections: (1) data collection: occurrence records of kiwifruit (*Actinidia* spp) species and environmental factors (bioclimatic, topographic, and soil factors) were obtained; (2) data preprocessing: occurrence records were converted to CSV files, samples that did not match environmental factors were filtered, environmental factors were all at 30” resolution, and input factors with the least amount of redundancy were filtered through correlation analyses; (3) parameter optimization and model setup: The ‘ENMevel’ package in R was utilized to select the optimal parameters from 72 candidate models, and subsequently, the MaxEnt model of kiwifruit species was established to predict the future period. And (4) Evaluation and analysis: Model accuracy was assessed using the AUC (Area Under the Curve) metric. Key environmental factors were identified through contribution ratios, response curves, and jackknife tests. Additionally, the SDMTool was employed to analyze changes in habitat suitability zones and their center of mass over time. A multi-factor correlation analysis was conducted to explore the relationship between suitability areas and kiwifruit yield trends.

### 2.2 Study areas and species occurrence data

**Figure 2:**
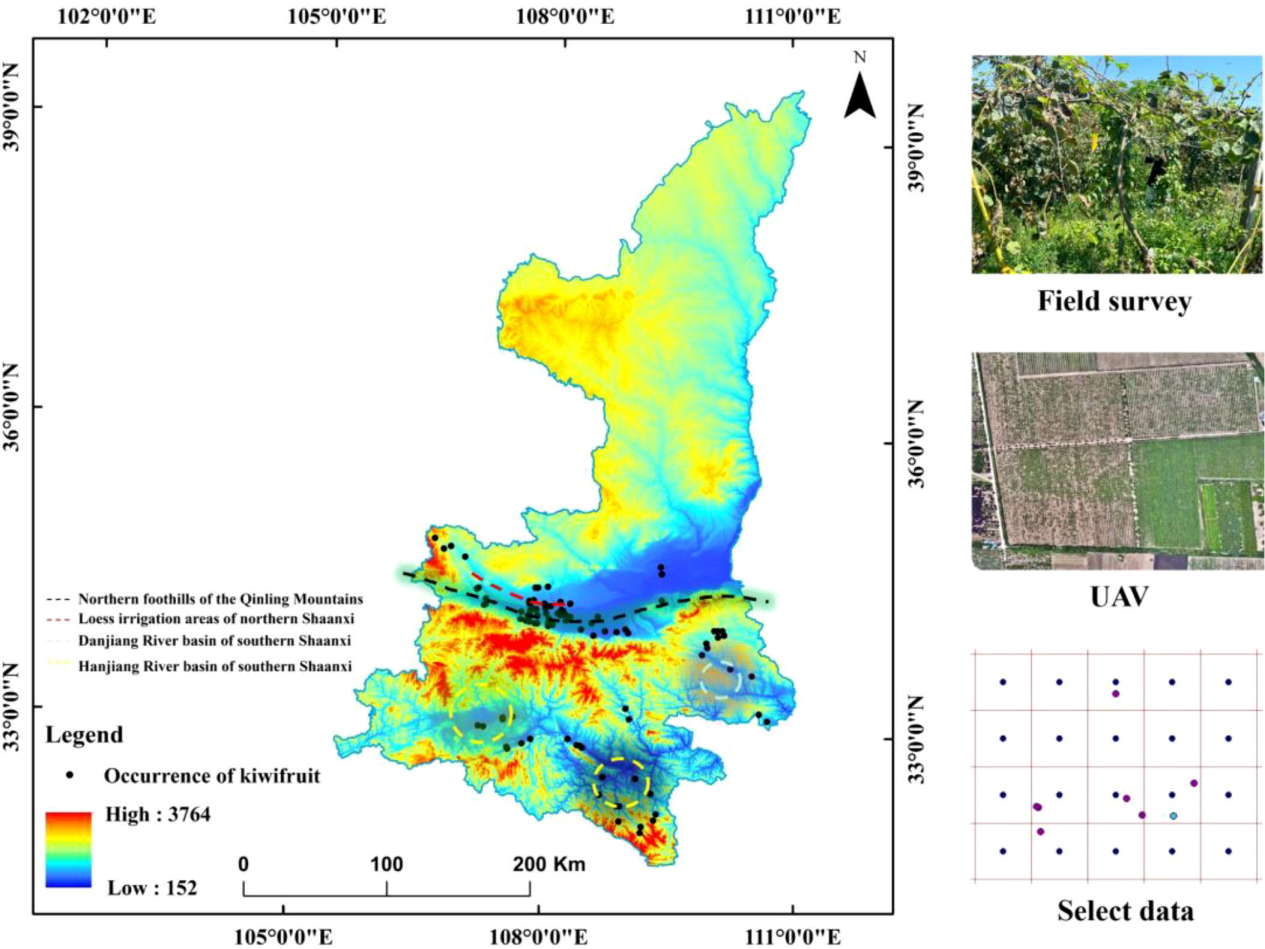
Distribution of kiwifruit occurrences.

Shaanxi Province is located in the transitional zone between the northern and southern climates in China, spanning various landforms such as plateaus, mountains, and plains. The annual temperature variation ranges from 31.57°C to 35.62°C, and the altitude gradient covers from 6 meters to 678.03 meters (Zhang and Li, 2022). This environmental heterogeneity poses strict requirements on the climatic adaptability of kiwifruit. Research has shown that extreme high - temperature stress can directly inhibit the physiological functions of plants (Raza et al., 2023), causing photosynthetic products to be preferentially allocated to vegetative growth and significantly reducing the carbohydrate and vitamin C content in fruits. Meanwhile, the imbalance in the precipitation pattern increases the risk of soil anoxia through extreme rainfall events (Furtak and Wolińska, 2023), threatening root respiration and leaf photosynthetic efficiency. In terms of hydrological conditions, during the fruit - swelling period, the soil water content needs to be maintained in the appropriate range of 6508.30 - 11332.82 mm. However, the kiwifruit’s intolerance to waterlogging (Li et al., 2021) requires that the planting area has efficient drainage capacity to avoid metabolic inhibition caused by root anoxia. Analysis of soil characteristics shows that kiwifruit in Shaanxi Province is concentrated in loam areas. Its pore structure can ensure the exchange efficiency of air and water and maintain moderate water - holding capacity. The soil organic carbon content and PH further screen suitable habitats by regulating nutrient availability. The distribution data of kiwifruit in Shaanxi Province (n = 259) were collected through the Global Biodiversity Information Facility (GBIF), the Chinese Virtual Herbarium (DHC), and field surveys. To ensure the temporal consistency between the species distribution data and the environmental data (from 1970 to 2020), orchard data recorded during this period and from orchards with trees over five years old were selected (n = 207). The ENMTools software was used to eliminate spatially redundant records at a resolution of 1 km. Finally, 99 valid distribution points (Field survey data is 3.03%) were obtained for model training (**Figure 2**).

### 2.3 Environment variable filtering

**Figure 3:**
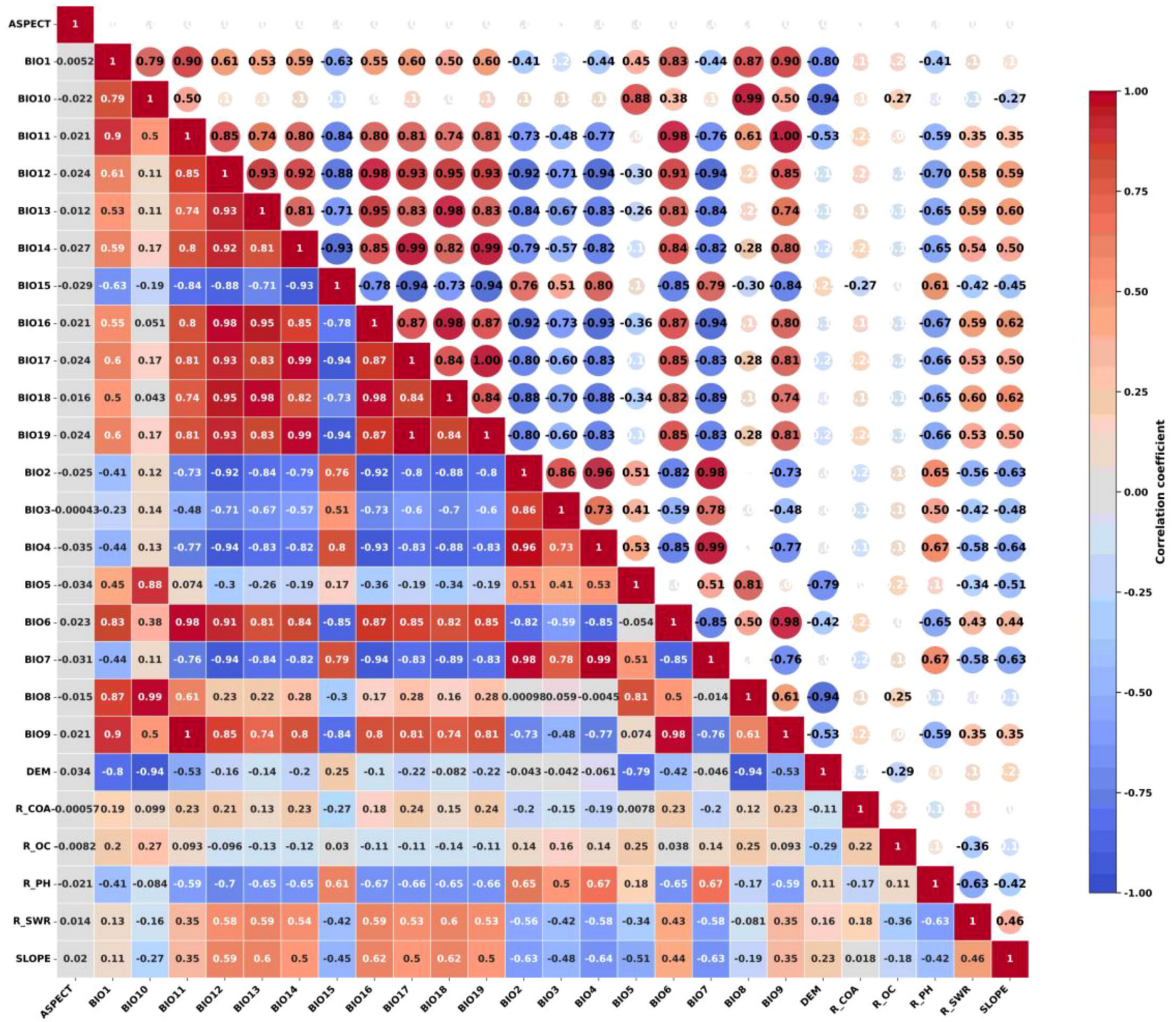
Matrix of correlation coefficients for 26 environmental factors.

The study encompassed a total of 26 environmental factors, comprising 19 climatic factors, 4 soil factors within the lower soil layer, and 3 topographic factors (**Table 1**). The climate data were obtained from the World Climate Database (http://www.worldclim.org/), and the baseline climate (1970-2020), future climate scenarios 2050 (2041-2060) and 2070 (2061-2080) from the BBC_CSM climate modelling system were used in this study. The future climate scenarios selected were SSP126, SSP245, SSP370, and SSP585, four emission models containing 19 bioclimatic factors important to species distribution and elevation data, with a spatial resolution of 30″(approximately 1km×1km). Slope and aspect data were calculated from elevation data. Slope and aspect data were calculated from elevation data. Soil data were obtained from the Food and Agriculture Organization of the United Nations (https://www.fao.org/) HWSDv1.0. The survival range of the kiwifruit root system is considered to be between 30-60cm, thus making it suitable for growth in slightly acidic soil with deep, fertile and loose layers and adequate drainage. It is imperative that the soil is rich in organic matter to ensure the full extension of the root system and the absorption of nutrients. The selection of the soil property data of the layer, including soil pH, soil coarse grain size, soil organic carbon content, and soil water content, was made with a spatial resolution of 30’ (approximately 1km×1km). The vector boundary maps involved in the study were obtained from the standard map service system (http://bzdt.ch.mnr.gov.cn/). Through the results of a single simulation test, the environmental factors that contributed less than 0.5% in the percentage contribution of environmental factors in the MaxEnt model output were excluded. The correlation between the factors was also analyzed using correlation, and the heat map of correlation coefficients (**Figure 3**) was used to retain factors with correlation coefficients less than 0.80. Finally, 11 environmental factors were selected as the dominant environmental factors in this study.

**Table 1:** Environmental factors predicted by the model.

No.	Factor description	Abbreviation	Unit
1	Annual Mean Temperature	BIO1	°C
2	Mean Diurnal Range [Mean of monthly (max temp - min temp)]	BIO2	°C
3	Isothermality (BIO2/BIO7) (×100)	BIO3	×100
4	Temperature Seasonality (standard deviation ×100)	BIO4	×100
**5**	**Max Temperature of Warmest Month**	**BIO5**	**°C**
6	Min Temperature of Coldest Month	BIO6	°C
**7**	**Temperature Annual Range (BIO5-BIO6)**	**BIO7**	**°C**
8	Mean Temperature of Wettest Quarter	BIO8	°C
**9**	**Mean Temperature of Driest Quarter**	**BIO9**	**°C**
10	Mean Temperature of Warmest Quarter	BIO10	°C
**11**	**Mean Temperature of Coldest Quarter**	**BIO11**	**°C**
12	Annual Precipitation	BIO12	mm
13	Precipitation of Wettest Month	BIO13	mm
14	Precipitation of Driest Month	BIO14	mm
15	Precipitation Seasonality (Coefficient of Variation)	BIO15	×100
16	Precipitation of Wettest Quarter	BIO16	mm
17	Precipitation of Driest Quarter	BIO17	mm
18	Precipitation of Warmest Quarter	BIO18	mm
19	Precipitation of Coldest Quarter	BIO19	mm
**20**	**Soil PH (H_2_O)**	**R_PH**	**-log(H+)**
**21**	**Soil Water Regime**	**R_SWR**	**-**
**22**	**Soil Organic Carbon**	**R_OC**	**%**
**23**	**Soil Coarse**	**R_COA**	**%**
**24**	**DEM**	**-**	**m**
**25**	**SLOPE**	**-**	**-**
**26**	**ASPECT**	**-**	**(°)**

*Factors in bold are for the final predictive model.

### 2.4 MaxEnt model and parameter optimization

#### 2.4.1 MaxEnt model

The Maximum Entropy (MaxEnt) model is a probabilistic method used for species distribution modeling. It estimates the most likely distribution of a species based on environmental conditions and presence-only data (Phillips et al., 2017). In actual species surveys, the recorded data on species occurrence is often limited, yet MaxEnt has been shown to perform better even with smaller sample sizes. The MaxEnt principle has attracted considerable attention in the field of machine learning due to its effective handling of complex and incomplete data. Specifically, in the context of species distribution modelling, it predicts the areas where species are likely to be distributed by analyzing known species presence points and environmental factors. By maximizing the conditional entropy, the MaxEnt model is able to make the least biased prediction of the distribution of a species with limited information, with the following formula:


$$P\left(y|x\right)=\frac{1}{Z\left(x\right)}exp\sum_{i}\lambda_{i}f_{i}\left(x,y\right)$$

In Eq. P(y│x) denotes the probability that species y exists at location x, $f_{i}\left(x,y\right)$ denotes the relationship between input x and input y in the ith eigenfunction, $\lambda_{i}$ is the weighting factor of $f_{i}\left(x,y\right)$ . Z(x) is the normalization factor that ensures that the probabilities sum to one.

#### 2.4.2 Parameter Localization

In the MaxEnt model, the feature combination (FC) (Zhao et al., 2021) and the regularization multiplier (RM) (Yan et al., 2021) are key hyperparameters that affect the model’s performance. The FC parameter characterizes the species - environment relationship by selecting the types of feature functions (linear-L, quadratic-Q, hinge-H, product-P and thresholding -T, or their combinations) (Sutela et al., 2021). The RM parameter regulates the regularization strength through the penalty weight coefficient λ_i (Zhao et al., 2024), thus preventing overfitting during the model optimization process. It is evident that different types of eigenfunctions are capable of capturing the complex and factor relationship between environmental factors and species presence with greater efficacy. Conversely, the regularization multiplier (RM) is instrumental in averting overfitting by calibrating the intensity of regularization through the imposition of penalties on the weight coefficients, λ_i, during the optimization process of the model (Shi et al., 2021). It is evident that judicious calibration of the model’s two parameters, FC and RM, enhances its predictive accuracy and generalization capability, particularly in the context of novel data. This enhancement in prediction ability is accompanied by an improvement in the capture of key environmental factors, leading to a reduction in sensitivity to noisy or non-representative data.

**Figure 4:**
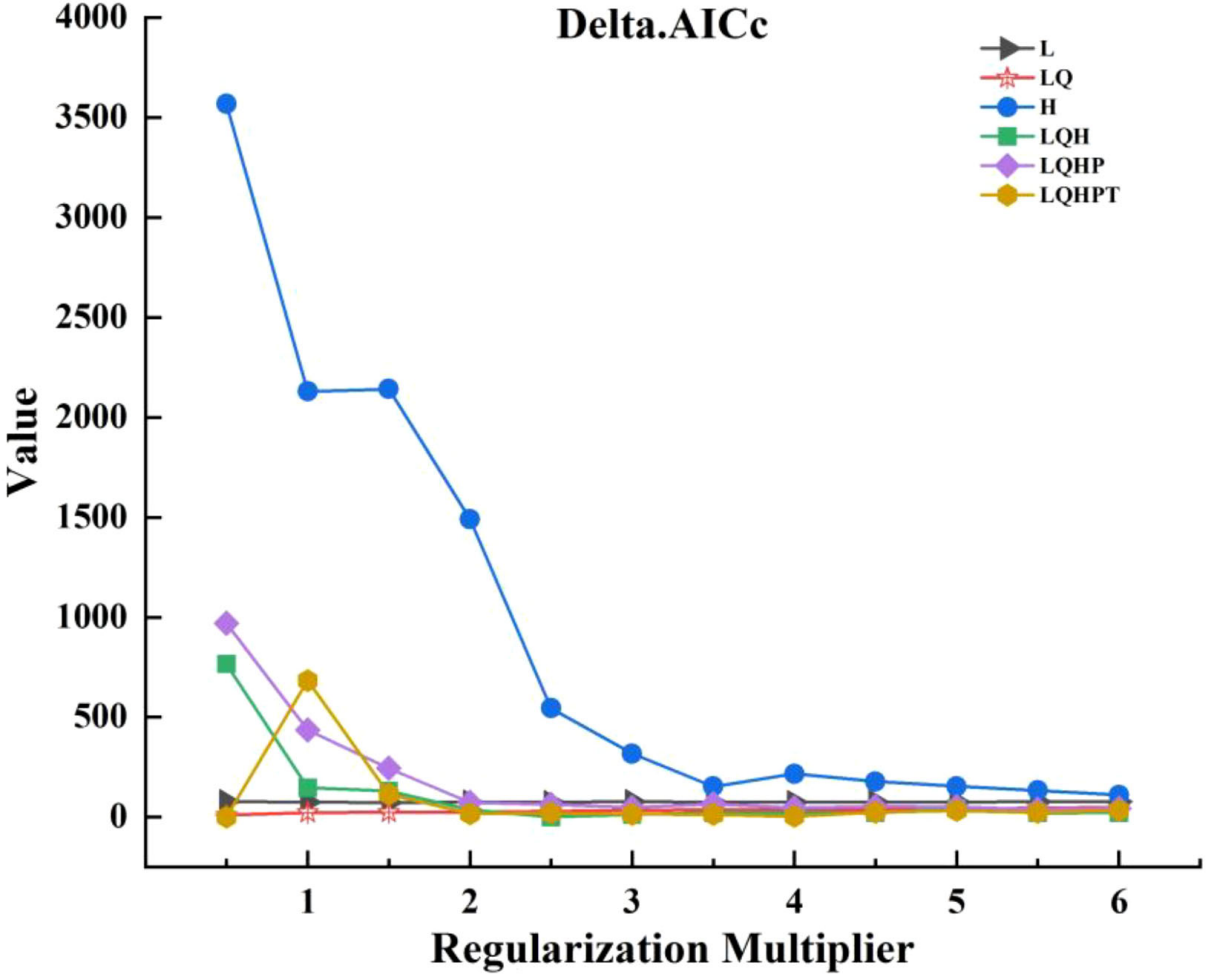
Optimization results of MaxEnt model parameters.

The optimal parameters were selected using the ENMeval package, and a total of 72 candidate models were generated by combining all six combinations of the 12 RM settings (from 0 to 6, with a spacing of 0.5) with the five FC class settings (Kang et al., 2025). The selection of the model was prioritized according to statistical significance, leakage rate and the Akaike Information Criterion (AICc). Initially, candidate models were screened to retain statistically significant models. Subsequently, the omission rate criterion was employed (i.e., omission rate<5%). Finally, among the statistically significant and low omission candidate models, the model with the lowest Delta.AICc value was selected. The combination of FC and RM at the 2.5 level was ultimately selected for the construction of the optimal model (**Figure 4**).

### 2.5 MaxEnt model fitting and kiwifruit suitability zone delineation

The MaxEnt model is employed to predict the potential geographic distribution of the species, utilizing distribution data and environmental factor data to construct a relationship model. This model is then used to analyze the distribution suitability. A random selection of 25% of the distribution data was designated as the test set, with the remaining 75% of the distribution data being utilized as the training set for the construction of the kiwifruit habitat model with 10 operations. The contribution of environmental factors to species distribution was calculated, and response curves were plotted to analyze the environmental factors affecting species distribution and their thresholds. The area under the ROC curve, i.e. the AUC value was plotted for the simulation accuracy test of the model, and its magnitude gives a better indication of the accuracy of the simulated values of the model. An AUC of 0.5 to 0.6 indicates a poor simulation, 0.6 to 0.7 indicates a fair simulation, 0.7 to 0.8 indicates an accurate simulation, 0.8 to 1.0 indicates a very accurate simulation (Chicco and Jurman, 2023).

The ASCII data obtained from the model were converted to raster data using ArcGIS, and the presence probability P was reclassified. The minimum training presence logistic threshold (Mtp) in the MaxEnt model was utilized to ascertain the suitability of the distribution. The criteria for delineating the suitable distribution area are as follows: P<0.1 for non-suitable area, 0.1≤P<0.3 for low suitable area, 0.3≤P<0.6 for medium suitable area, and 0.6≤P for high suitable area.

## 3 Results and analyses

### 3.1 Model accuracy and current distribution

**Figure 5:**
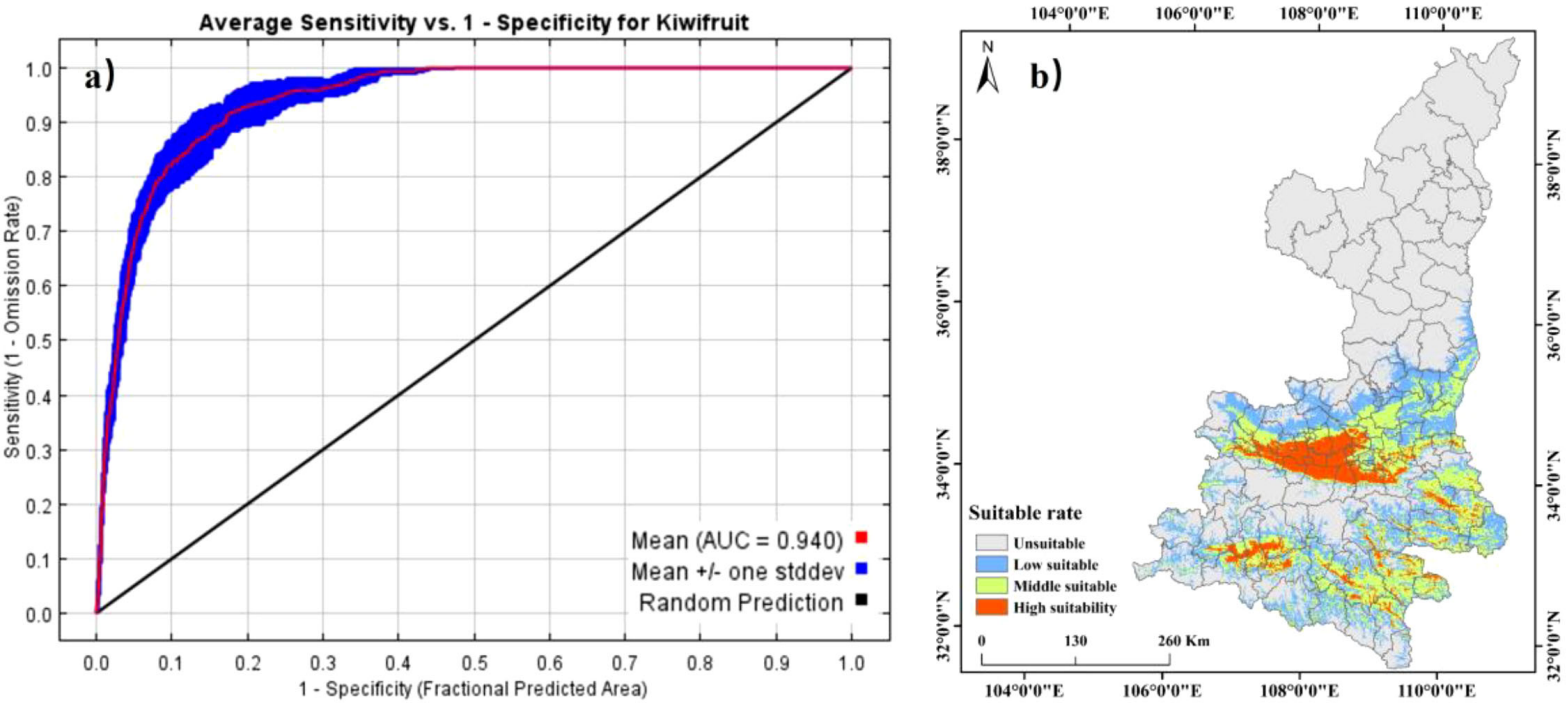
Validation of ROC curves **(a)** and distribution of kiwifruit suitable areas **(b)** for MaxEnt model predictions.

The MaxEnt model performance was evaluated using the Area Under the Curve (AUC) metric (**Figure 5**). The optimized model achieved an average AUC of 0.940 for training data and 0.918 for test data, reflecting a 7.55% improvement over the original model (**Table 2**). These results confirm that the model provides high predictive accuracy for kiwifruit habitat distribution. Based on the suitability classification, kiwifruit habitats were reclassified into four classes (**Figure 5**), with a suitability index of 30 per cent as the threshold, and areas with a suitability index greater than 30 per cent were classified as suitable habitat for kiwifruit. Suitable habitats for kiwifruit plants were mainly located at the foot of the foothills at low and middle elevations. The total area of suitable habitats was 83,000 km², of which 13,100 km² were highly suitable habitats and 32,600 km² were moderately suitable habitats. These habitats are mainly located in the northern foothills of the Qinling Mountains, the loess irrigation areas of northern Shaanxi, the Danjiang River basin of southern Shaanxi, and the Hanjiang River basin of southern Shaanxi, including mainly the cities of Weinan, Hanzhong, Xi’an and Baoji.

**Table 2:** Comparison of accuracy of different parameter combinations.

Style	Regularization multiplier	Feature combination	Delta.AICc	AUC
prototype	1	LQHPT	683.6981	0.8534
post-optimization	2.5	LQH	1.4366	0.9178

### 3.2 Critical environmental factors

**Figure 6:**
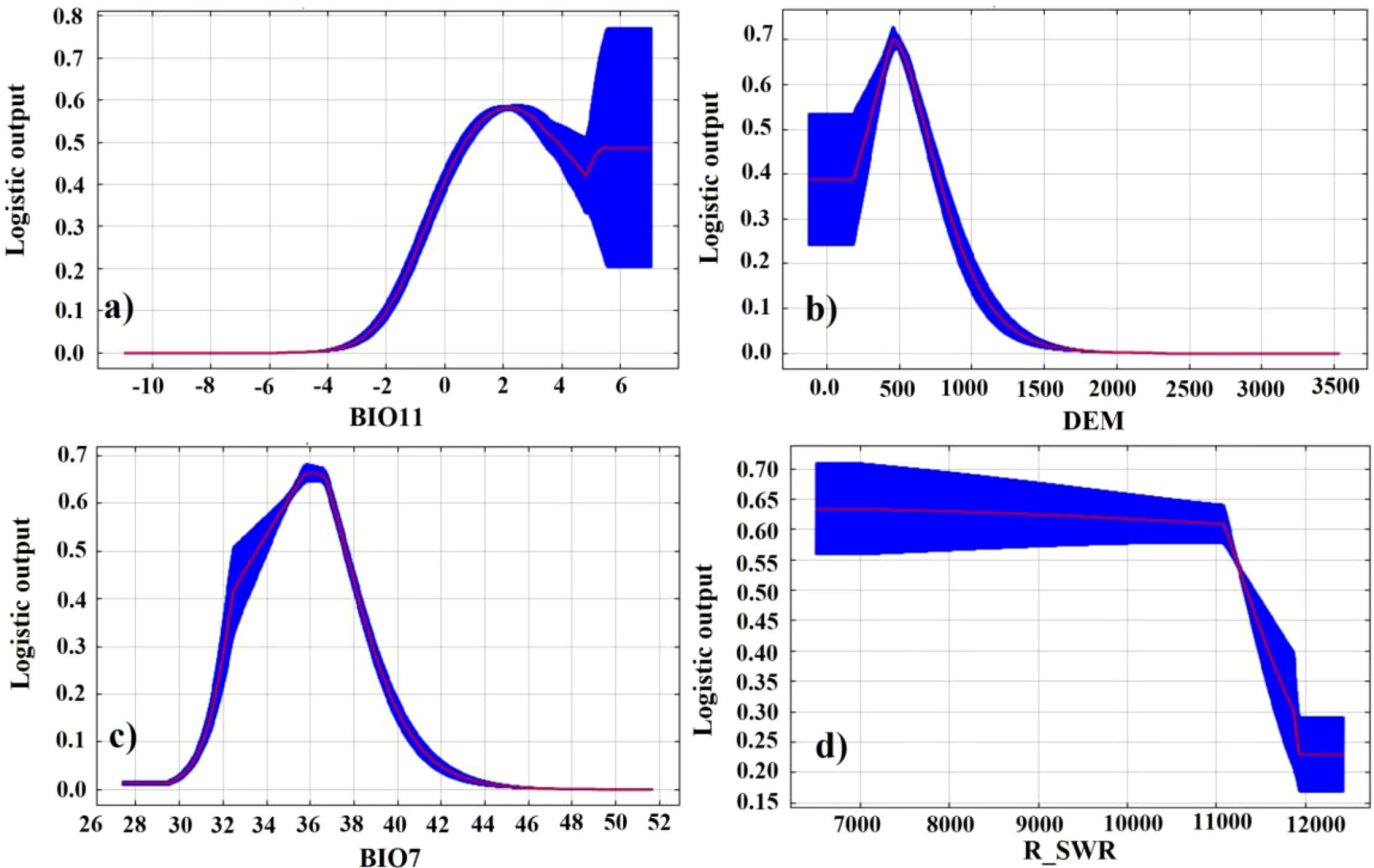
Corresponding curves of probability of occurrence of kiwifruit species and key environmental factors: **(a)** BIO11, **(b)** DEM, **(c)** BIO7, **(d)** R_SWR.

Considering the contribution of environmental factors, the variations in the probability of kiwifruit presence in response to environmental factors were analyzed, as well as the importance of each environmental factor in MaxEnt, suitable environments and key factors affecting kiwifruit growth. As illustrated in **Figure 6**, graphs and margins of error are integrated for the response curves between each factor and the probability of kiwifruit presence. The probability of kiwifruit presence varies parabolically with the range of coldest season mean temperature, elevation, and annual temperature. The probability of occurrence of kiwifruit plants was found to be 0.58 at the coldest quarterly mean temperature of 2.3°C, representing the optimal winter temperature conditions for kiwifruit plants. At an altitude of 483 m, the probability of kiwifruit plant occurrence reached 0.69, representing the optimal altitude conditions. Conversely, the probability of kiwifruit occurrence was 0.64 when the annual temperature range was 32°C, signifying the optimal annual temperature difference. Conversely, an increase in soil moisture content was observed to result in a decline in the probability of kiwifruit plant occurrence.

**Figure 7:**
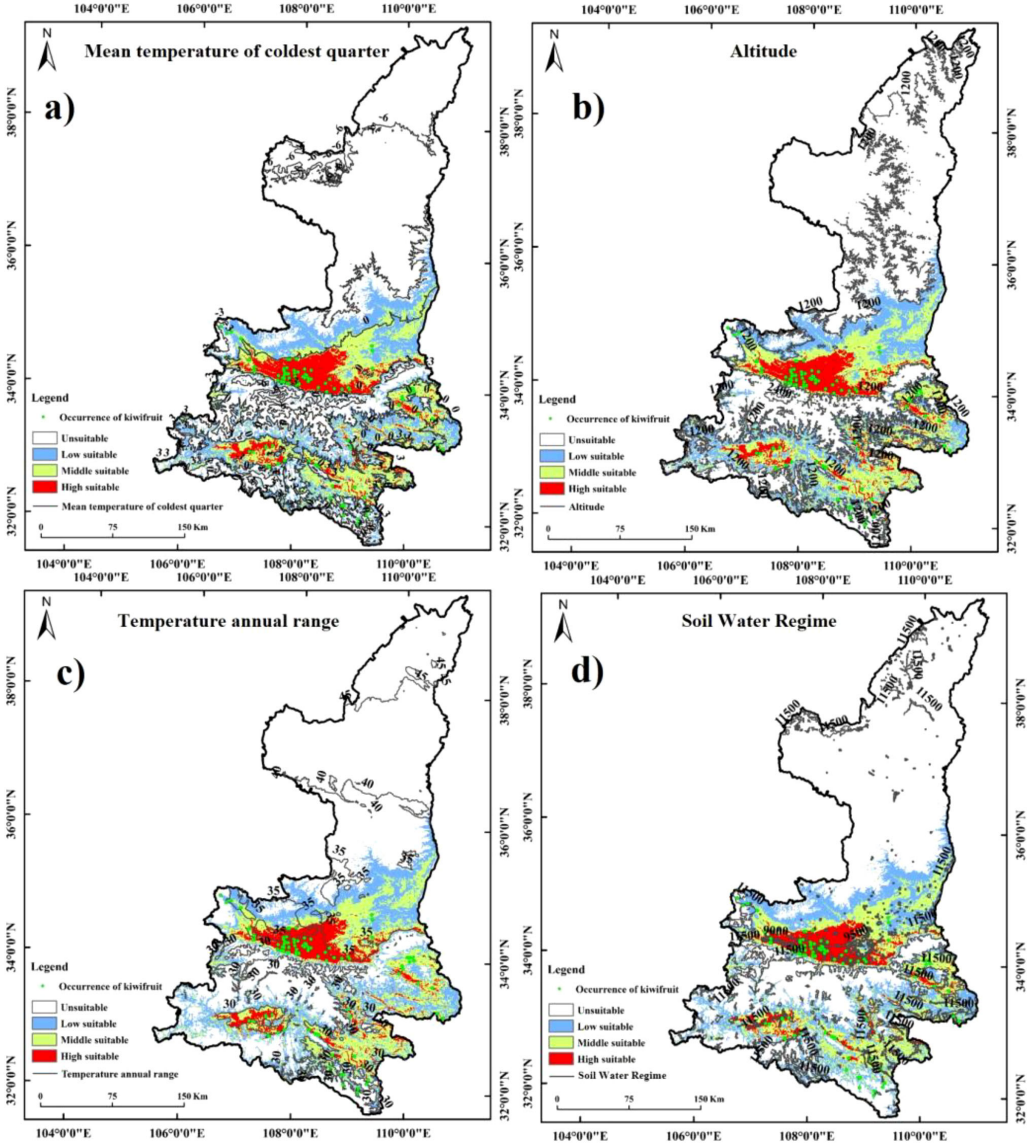
Synthesis of kiwifruit sample site distribution, habitat suitability zones and contours of environmental factors: **(a)** Mean temperature of coldest quarter, **(b)** Altitude, **(c)** Temperature annual range, **(d)** Soil Water Regime.

Furthermore, the most significant factors affecting the spatial distribution of kiwifruit were BIO11 (32.81%), DEM (31.04%), BIO7 (9.18%), and r_swr (9.06%), as demonstrated in **Table 3**. The cumulative contribution of the first four factors was more than 80%. The thresholds and means of the primary environmental factors were derived using a probability threshold of >0.5 for suitable kiwifruit habitat. As illustrated in **Table 3**, values above 0.5 are considered optimal in terms of applicability probabilities. For instance, the mean temperature of the coldest quarter ranged from 0.69°C to 3.83°C, with a mean temperature of 2.26°C. The range of elevation was from 282.36 m to 678.03 m, with a mean elevation of 480.20 m. The distribution of kiwifruit sample sites was superimposed with the habitat suitability zones and contours of the environmental factors. This showed that the distribution points mainly occurred within the optimal thresholds for the environmental factors (**Figure 7**).

**Table 3:** Contributions of major environmental factors and suitability thresholds.

Factor	Percentage of variance (%)	Cumulative percentage (%)	Threshold values (SI >50%)	
			Range	Mean
BIO11	32.8136	32.8136	0.69°C~3.83°C	2.26°C
DEM	31.0428	63.8564	282.36m~678.03m	480.20m
BIO7	9.1847	73.0411	31.57°C~35.62°C	33.60°C
R_SWR	9.0558	82.0969	6508.30~11332.82	8920.56

**Figure 8:**
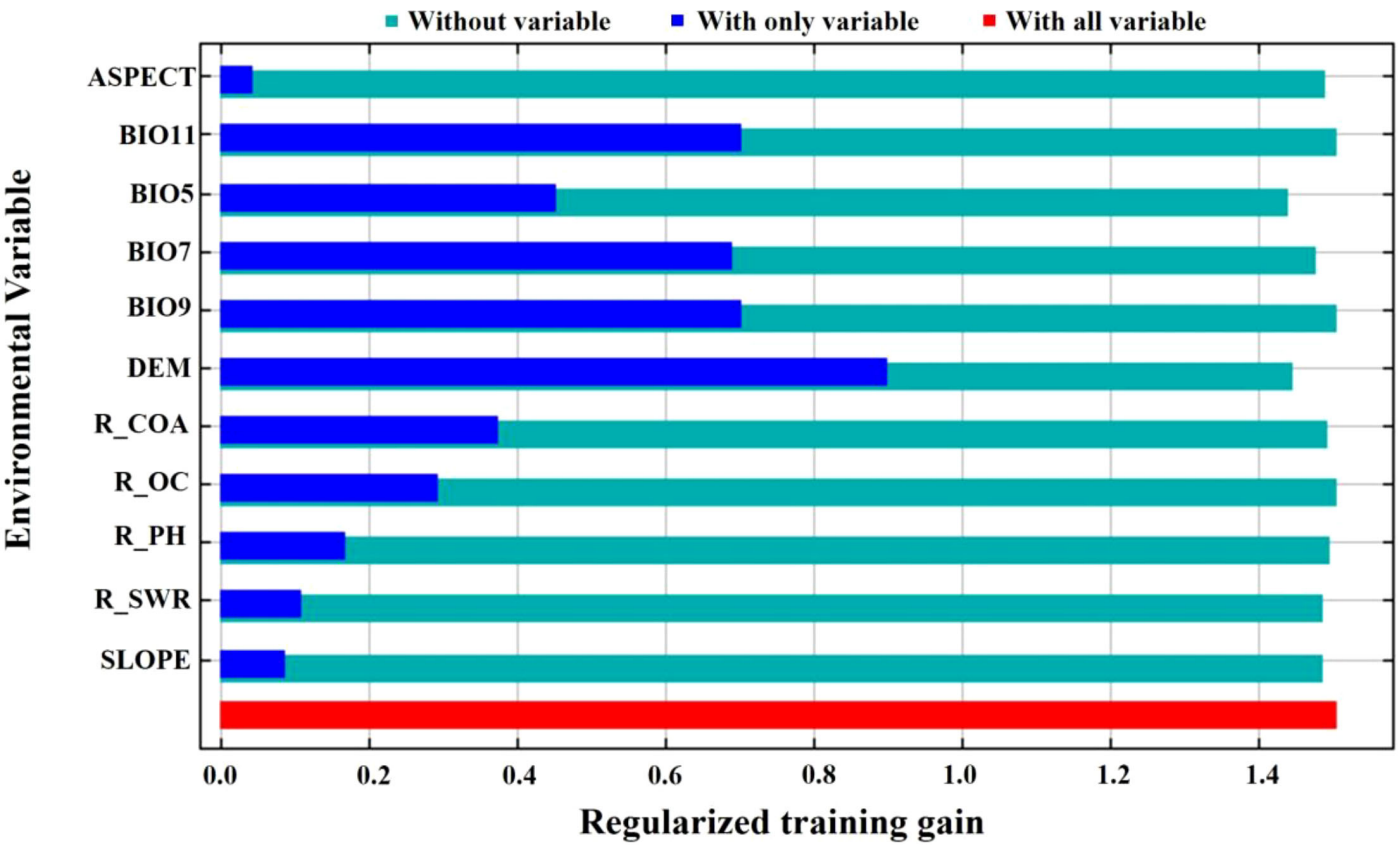
Importance of environment factors in Jackknife in MaxEnt.

The results of the importance of the environmental factors and the regularized training gain in MaxEnt are demonstrated in **Figure 8**. The results of the folding knife test demonstrated that the elevation data exhibited the most significant gain in the model, and the MaxEnt model, which disregards the elevation drive, resulted in a substantial decrease in gain. When a single factor was considered, elevation, the coldest quarterly mean temperature, the annual temperature range, and the driest quarterly mean temperature exhibited higher values of normalized gain, strongly suggesting their significant contribution to model performance.

### 3.3 Potential distribution of Shaanxi kiwifruit under future meteorological scenarios

**Figure 9:**
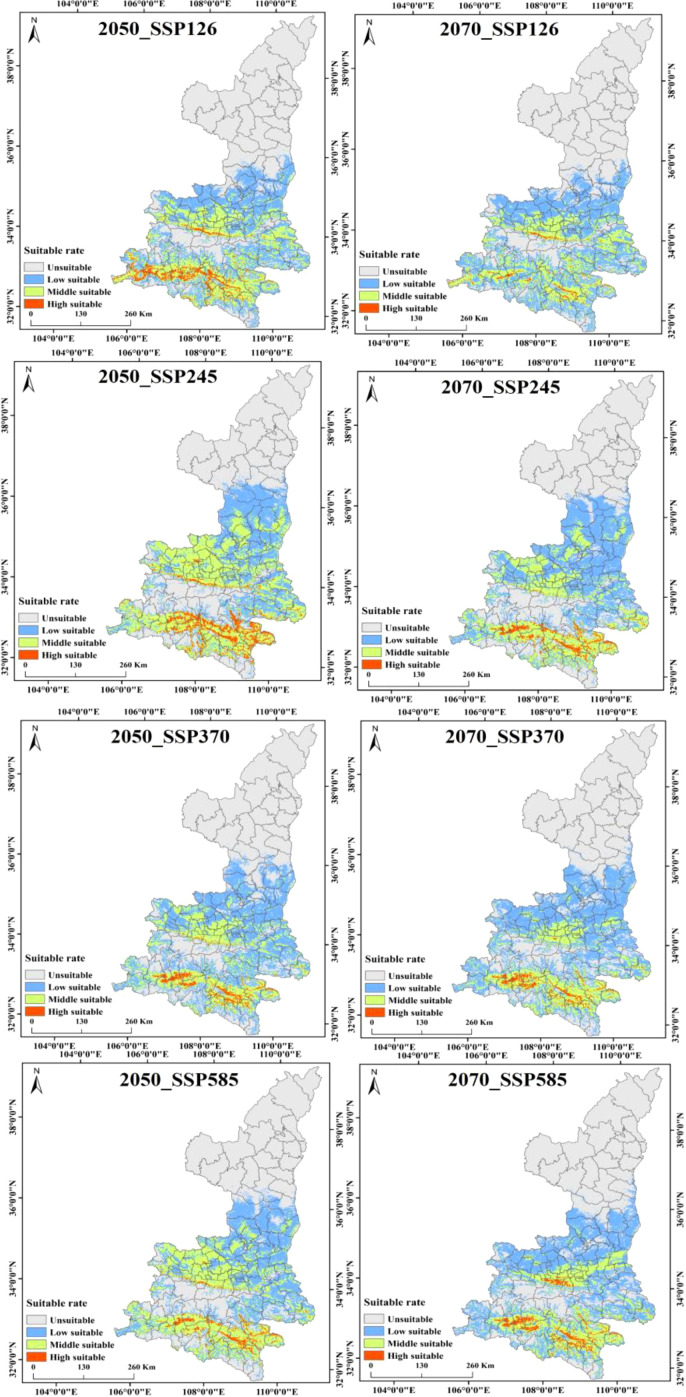
Habitat suitability of Shaanxi kiwi under four shared socio-economic pathways in the 2050 and 2070.

**Figure 10:**
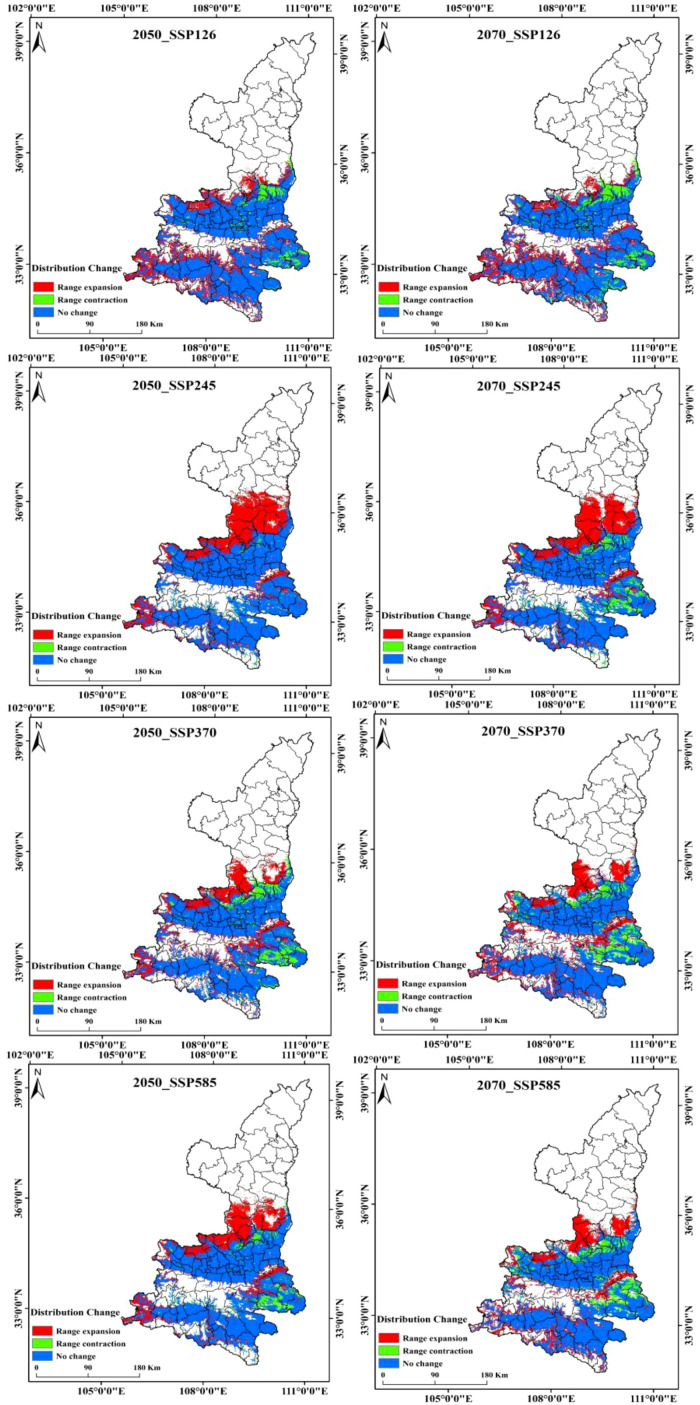
Changes in suitable areas for kiwifruit under different climate scenarios.

Kiwifruit is predominantly distributed near the foothill margins, one of the most sensitive areas to climate change. Therefore, the MaxEnt model was used to project potential suitable areas for kiwifruit in the 2050 and 2070 under scenarios SSP126, SSP245, SSP370 and SSP585. **Figure 9** shows the spatial distribution of future kiwifruit suitability zones, categorized into high, medium and low suitability zones. By the 2070, significant fragmentation of kiwifruit suitable habitat is expected in all scenarios. Geographically, the distribution of kiwifruit in Shaanxi Province gradually expands to higher latitudes and altitudes as time and radiation intensity increase, and highly suitable areas recede to the south, focusing mainly on Chenggu and Hanbin in the Han River basin of southern Shaanxi. Under the SSP126 scenario, highly suitable areas for kiwifruit in Shaanxi Province will gradually disappear, and under the SSP370 scenario, the northern foothills of the Qinling Mountains, the Weibei Plateau Irrigation Area, and the Danjiang River Basin in southern Shaanxi gradually degenerate into low suitable areas. In the long term, the area of suitable habitat for kiwifruit is expected to rise and then fall under the influence of relatively low social development and emission trajectories. The area of low suitability areas will continue to rise, the area of medium suitability areas will rise and then fall, and the area of high suitability areas will continue to fall. The area of suitable habitat for kiwifruit is projected to continue to rise under relatively high development and emission trajectories. Under all scenarios, the area of suitable habitat disappears mainly in Shangzhou District in the Danjiang River Basin in southern Shaanxi Province, and expands mainly in the marginal parts of the current suitable habitat, mainly in Huangling and Xunyi.The area of suitable habitat under the SSP245 emission scenario would increase at a higher rate in the 2050, and the degraded area would also gradually increase by the 2070. Under the SSP370 and SSP585 emission scenarios, the area of extended relatively less point habitat is expected to decrease significantly, as shown in **Figure 10**. This suggests that lower and higher SSP and emission pathways may have significant negative impacts on kiwifruit habitat.

**Figure 11:**
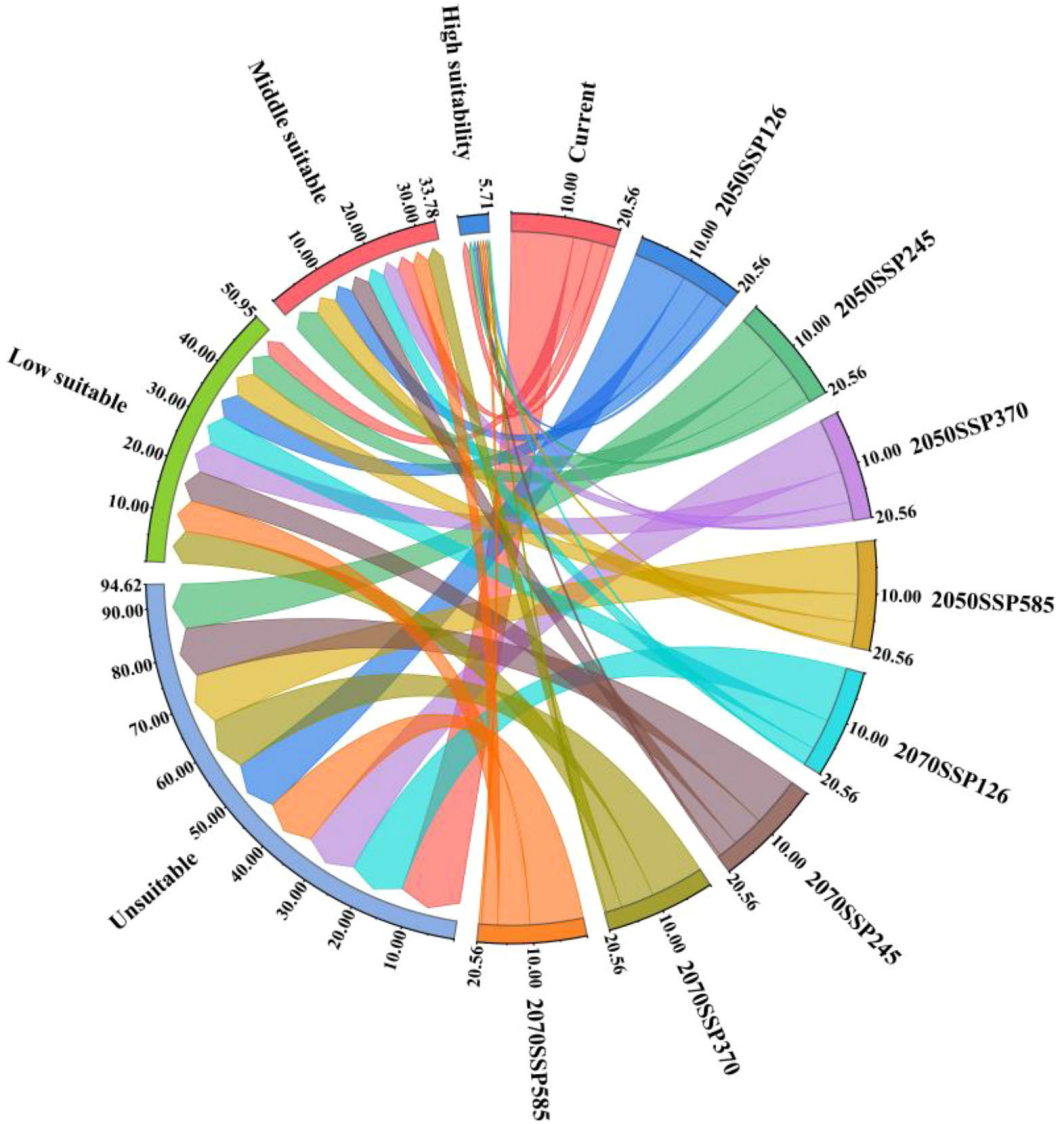
Changes in suitable area of kiwifruit with time under different SSP scenarios.

As illustrated in **Figure 11**, the temporal progression of the suitable area for kiwifruit cultivation is delineated according to specific SSP scenarios. It is evident that the area of highly suitable areas is in continuous decline, with the high emissions scenarios (SSP370, SSP585) resulting in a more than 50% reduction in the area of highly suitable areas by the 2050, from 13,100 km^2^ to 4600 km^2^ and 5200 km^2^, respectively. The SSP126 emissions scenario and the SSP245 emissions scenario demonstrate a decline of 46.56% and 25.95%, respectively. By the 2070, the area of the high suitability region for the four emission scenarios (126, 245, 370, and 585) decreases to 0.27, 0.55, 0.42, and 0.5 million square kilometers, respectively. For the medium suitable area, the area first increases and then decreases, and by the 2050, the area of medium suitable area under the SSP245 emission scenario increases from 32,600 km^2^ to 54,200 km^2^, which is an increase of more than 66.26%. By the 2070, the area of the medium suitable area under the SSP370 and SSP585 emission scenarios decreases to less than the area of the contemporary medium suitable area, and the area of the medium suitable area under the other two emission scenarios remains higher than the area of the contemporary medium suitable area. Conversely, the area of low suitability is projected to increase under all emission scenarios. By the 2070, the area of low suitability zones for the four emission scenarios (126, 245, 370, and 585) is 606, 626, 659, and 63,200 square kilometers, respectively.

### 3.4 Shaanxi kiwifruit center of mass movement under future meteorological scenarios

**Figure 12:**
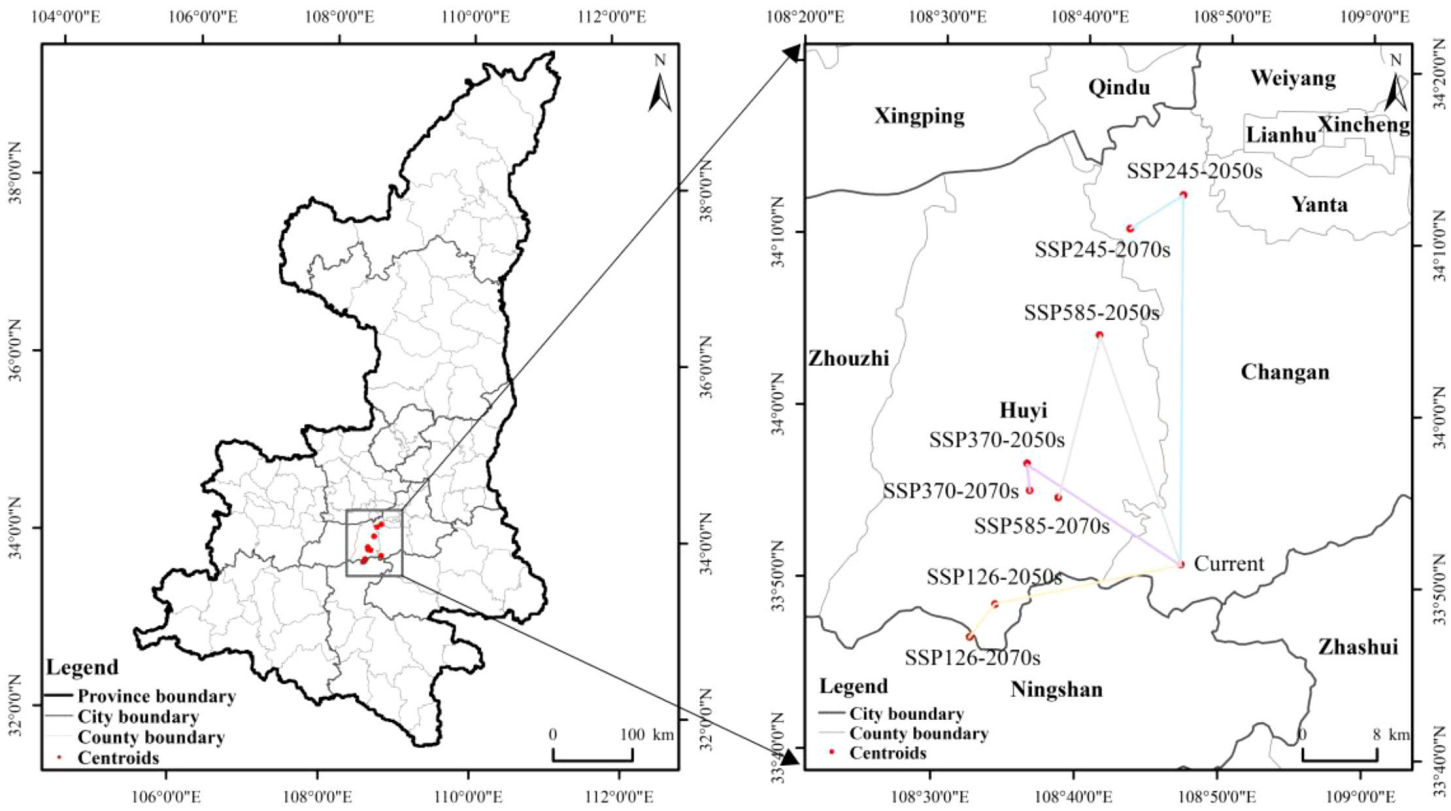
Changes in center of mass in kiwifruit suitability zones under future climate scenarios.

ArcGIS was utilized to calculate the location and directional movement of the center of mass of the kiwifruit suitability area for each scenario (**Figure 12**). The current kiwifruit suitability center is located in Chang’an District at coordinates 108.786°E, 33.853°N. In the SSP126 scenario, the center of distribution of kiwifruit is projected to move in a southwesterly direction through Huyi until it reaches Ningshaan County in 2080 at coordinates 108.571°E, 33.810°N. In the SSP245 scenario, the center is projected to move in a southwesterly direction from 2040 to 2060, then in a northeasterly direction, and finally, from 2060 to 2080, it is projected to move in a southwesterly direction to the northern part of Chang’an District with coordinates 108.719°E, 34.177°N. In the SSP370 scenario, the center of mass is projected to move in a northwesterly direction from 2040 to 2060; however, it is predicted to move south-eastward to reach Huyi by 2060 to 2080, with coordinates 108.609°E, 33.921°N. In the SSP585 scenario, the center of mass is projected to move in a north-westerly direction from 2040 to 2060, and then move south-westerly to reach Huyi, with coordinates of 108.642°E, 33.915°N. In summary, based on the projected future climatic conditions, the center of distribution of kiwifruit is expected to shift slightly northwards and westwards with time.

## 4 Discussions

### 4.1 Consistency of climate suitability zones and yield in kiwifruit

**Figure 13:**
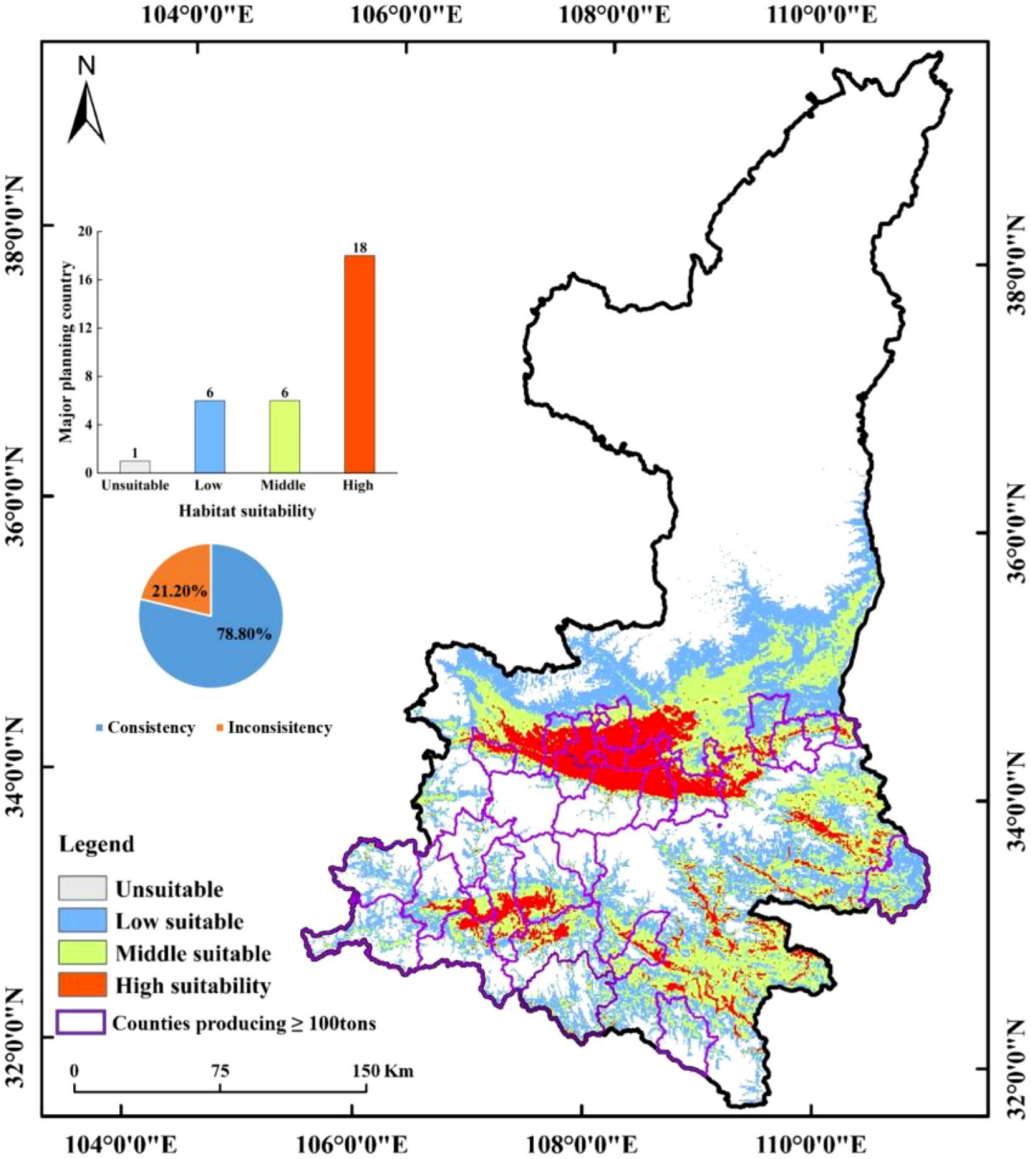
Regional map of the distribution of major producing counties with annual production greater than 100 tonnes.

As demonstrated in **Figure 9**, the future primary producing countries continue to be classified as suitable zones. In conjunction with the feasibility of data collection, 31 main producing counties with average kiwifruit yield data of more than 100 tonnes (annual production) from 2018 to 2020 were collected, and the climate suitability zone hierarchy map and the main kiwifruit producing counties were superimposed and analyzed, so as to count the consistency of the main kiwifruit producing counties with the kiwifruit climate suitability zones simulated in the model, as shown in **Figure 13**.The study revealed that the kiwifruit yields were situated within the medium and high suitability zones. The analysis indicated that six and 18 counties, respectively, exhibited kiwifruit production in the medium and high suitability zones. Furthermore, the study found that the spatial overlap between kiwifruit yield and climate suitability zones was 78.80%, suggesting a high degree of congruence between the distribution of kiwifruit-producing counties and the climate suitability zones within the prevailing climatic conditions.

The average yield in the main producing counties was subjected to qualitative analysis, with the area of high suitability zone, the area of medium suitability zone, the area of low suitability zone, the maximum temperature of the hottest month (BIO5), the range of annual temperature variation (BIO7), the mean temperature of the driest season (BIO9) and the mean temperature of the coldest season (BIO11) all taken into consideration. Without considering technological progress, kiwifruit yield in the main producing counties was correlated with the area of the three types of suitable zones, with the strongest correlation being with the area of the medium suitable zone. It is predicted that the kiwifruit yield in the medium-suitable zone will undergo an initial increase, followed by a subsequent decrease over the next 40 years. Concurrently, the high-suitable zone is expected to experience a continuous decline. This analysis suggests that the fluctuations in the areas of the high-suitable zone, the medium-suitable zone, and the low-suitable zone will result in a factor kiwifruit yield in Shaanxi Province between 2040 and 2060. The impact of climate change is projected to be the predominant factor influencing this variability, though it is not anticipated to result in a substantial reduction in yield. However, a decline in yield is predicted to occur in certain areas during the 2060–2080 period.

### 4.2 Changes in spatial patterns

The study finds that kiwifruit distribution is mainly concentrated in southern Shaanxi and the Guanzhong region, with the most suitable habitats located in Weinan, Hanzhong, Xi’an, and Baoji. Shaanxi’s diverse geography creates a unique ecosystem, offering a range of ecological niches suitable for kiwifruit cultivation. Furthermore, the heterogeneous landscape of the Qinling Mountains has been shown to promote rapid species diversification. The foothills of the Qinling Mountains and their surrounding areas are biodiversity hotspots characterized by high species diversity, a large proportion of endemic species and unique plant communities (Huo et al., 2023). Projections indicate that kiwifruit’s suitable habitat will decline significantly under all four climate scenarios (SSP126, SSP245, SSP370, and SSP585) (Wang et al., 2024). The principal cause of this decline and fragmentation is believed to be climate warming.

In response to climate change, the center of kiwifruit cultivation is shifting geographically. The findings indicated that kiwifruit demonstrated a high degree of suitability in the Huyi District of Xi’an. The center of mass was located to the north of the current center of mass in different time periods and under different emission scenarios, except for the SSP126 emission. Consequently, the prediction indicates that the area of optimal conditions will coincide with the movement of the center of mass and expand in a northerly direction, influenced by future meteorological changes. The main manifestation of the kiwifruit suitable areas in Shaanxi Province responding to climate change is the movement towards higher latitudes and altitudes, which is consistent with the research results of other scholars (Wang et al., 2024; He et al., 2018). This finding also is consistent with the findings of related studies examining global warming that some species will migrate to higher dimensions and elevations (Zhang et al., 2018).

### 4.3 Variations in the effects of major environmental factors on kiwifruit

Kiwifruit sampling sites were largely within optimal environmental thresholds, confirming the high predictive accuracy of the model (**Figure 7**). The presence of abundant rainfall, complex topography, and diverse microclimates in Shaanxi has been demonstrated to facilitate optimal growth conditions for kiwifruit plants, with optimal growth observed at low to medium altitudes within zones characterized by low but not excessively cold winters. The study finds that kiwifruit thrives in cold-season temperatures between 0.69°C and 3.83°C, aligning with previous findings by GAO (Gao et al., 2022). The coldest seasonal mean temperature has a direct impact on the overwintering growth of kiwifruit, and low temperatures can cause frostbite and frost damage to kiwifruit plants (Liu et al., 2023). Altitude is recognized as one of the most significant environmental factors within the ecosystem, exerting a direct influence on the microenvironment of kiwifruit. Research has demonstrated that kiwifruit exhibits optimal productivity within the range of 20–610 meters above sea level. However, studies have also shown a decline in mean fruit physical traits with increasing altitude (Zenginbal and Ozcan, 2018). At higher altitudes, lower temperatures are observed, along with higher atmospheric pressure and radiation intensity. Kiwifruit exhibits a high degree of intolerance to direct sunlight, with intense summer light potentially leading to leaf scorching, particularly in high-temperature environments, thereby increasing susceptibility to sunburn (Li et al., 2022). Consequently, kiwifruit cultivation is predominantly situated in lower altitudes.

The four soil factors – soil pH, soil coarse grain size, soil organic carbon content, and soil water content – accounted for a relatively high proportion of soil water content in the modelled contribution (Zheng et al., 2023). It has been established that kiwifruit has an increased demand for water during the expansion period, and insufficient water will result in a decrease in the quality of the fruit per weight. On the other hand, since kiwifruit combines waterlogging intolerance, excessive soil moisture tends to inhibit root respiration and jeopardize fruit tree health (Torres-Ruiz et al., 2016). Soil coarse-grainedness determines the pore structure of the soil, and larger pores allow rapid passage of air and water, but the soil’s water-holding capacity is poor, so kiwifruit is mostly planted in loamy soils (Gentile et al., 2021).

### 4.4 Recommendations on the suitability of geographical indications for kiwifruit

The study analyzes the impact of climate change on kiwifruit’s geographical indications (GIs). The results indicate that the area deemed highly suitable for kiwifruit cultivation is projected to experience a substantial decline under various climate change scenarios. The modelled highly suitable acreage would disappear under the SSP126 scenario, and these findings can be used to develop scientific recommendations for kiwifruit planning and climate-smart agricultural policies in different regions in response to climate change. For instance, agricultural investors can utilize these predictions to assess potential future high-quality kiwifruit production areas, target investments, construct factories and arrange industrial layouts. It is important to note that the majority of kiwifruit growers in China are smallholder farmers who lack the financial resources and technical capabilities to mitigate the adverse effects of climate change on kiwifruit cultivation. Consequently, this study may provide recommendations for kiwifruit climate-smart cultivation, with the aim of adjusting long-term planting plans and optimizing cultivation patterns.

### 4.5 Research limitations

In order to comprehend the sensitivity and adaptation of crop ecosystems to climate change, it is necessary to consider the interactions between ecological and socio-economic factors (Tang et al., 2021). This study identified key environmental factors affecting kiwifruit, but limited data restricted analysis of biological interactions and human activity impacts. In future research, a micro perspective should be adopted to understand the interaction between factors and the suitability of kiwifruit GI cultivation by focusing on agricultural, policy and economic benefits.

## 5 Conclusions

The species distribution prediction framework constructed by this study based on the MaxEnt model optimized by the FC - LQH and RM 2.5 algorithms provides methodological innovation for the research on agricultural adaptability in ecologically sensitive areas. At the theoretical level, it reveals the response threshold of subtropical fruit trees to climate warming. It is found that the intensity of the effect of annual temperature difference fluctuations as a limiting factor is significantly higher than that of conventional variables in similar studies, which offers a new perspective for improving the parameter selection strategy of species distribution models (SDMs). In terms of practice, the study confirms that the spatial coupling degree between the main kiwifruit production areas in Shaanxi and the climatically suitable areas reaches 82.3%. It quantifies for the first time the critical maintenance effect of soil moisture (6508–11333 mm) on mountain orchards, establishing a replicable evaluation standard for climate - smart agricultural planning. In terms of knowledge innovation, through multi - scenario simulations, it is found that after 2060, the northern subtropical transition zone will exhibit the characteristic of “bimodal distribution of suitability”, that is, a complementary suitable belt will be formed between the high - altitude areas in the northern foothills of the Qinling Mountains and the low - altitude areas in the Hanjiang River Basin. This spatial compensation effect provides theoretical support for the climate adaptation strategies of mountain cash crops.

## Data Availability

Publicly available datasets were analyzed in this study. This data can be found here: http://www.worldclim.org/.
